# Report of the New York Ophthalmic Hospital

**Published:** 1860-03

**Authors:** 


*Seventh Annual Report of the Surgeons of the New York Ophthalmic Hospital.*—Drs. Stephenson and Garrish, the Attending Surgeons, report ten hundred and ten patients during the year 1859, and over 7,000 since 1852. The following are a list of the operations performed during the last winter, and, in some instances, a number of times, viz: Cataract, Strabismus, Pterygrum, Entropium, Ectropium, Trichiasis, Fistula Lachrymalis, Symblepharon, Staphyloma, and Extirpation of the Eye, (after the method of Mr. Critchett, of London;) also, Bowman's Operation for Catheterizing the Nasal Duct, by slitting up the lachrymal canal, with perfect success. The ophthalmic class of students and practitioners in attendance was larger than usual during the last session.

